# A real‐world, population‐based study of patterns of referral, treatment, and outcomes for advanced pancreatic cancer

**DOI:** 10.1002/cam4.1841

**Published:** 2018-10-30

**Authors:** Omar Abdel‐Rahman, Yuan Xu, Patricia A. Tang, Richard M. Lee‐Ying, Winson Y. Cheung

**Affiliations:** ^1^ Clinical Oncology Department, Faculty of Medicine Ain Shams University Cairo Egypt; ^2^ Department of Oncology University of Calgary Calgary Alberta Canada; ^3^ Department of Community Health Sciences University of Calgary Calgary Alberta Canada

**Keywords:** advanced pancreatic cancer, consultation, outcomes, referral, treatment

## Abstract

**Background:**

To describe patterns of referral, consultation, and treatment of advanced pancreatic cancer patients in a population‐based health care system and to evaluate the impact of these factors on outcomes.

**Methods:**

This is a retrospective analysis of population‐based cancer data from the province of Alberta, Canada. We analyzed patients diagnosed with either locally advanced or metastatic pancreatic adenocarcinoma from 2009 to 2016 and evaluated their patterns of referral to a cancer center, consultation with oncology, and treatment with active anticancer therapies. Logistic regression models were constructed to determine the factors associated with referral, late oncology assessment, and late receipt of treatment.

**Results:**

We identified 1621 pancreatic cancer patients. Median age was 70 years, 50% were men, and 51% had a Charlson index of 2+. Within this cohort, only 884 (54%) patients were referred to one of the provincial cancer centers. Adjusting for confounders in logistic regression models, older age and worse comorbidity scores were associated with nonreferral (both *P* < 0.01). In multivariable analysis among treated patients, the following factors were associated with improved overall survival, including younger age, earlier stage, and better comorbidity scores (all *P* < 0.01). Neither referral to consultation times nor consultation to treatment times correlated with outcomes. Importantly, nonreferred patients were more likely to use acute care services, including longer total duration of hospitalizations and more frequent visits with physician specialists.

**Conclusion:**

A significant proportion of patients with advanced pancreatic cancer were never referred to a cancer center. Nonreferred patients were more likely to utilize specific health care resources.

## 1 INTRODUCTION

Despite recent advances in cytotoxic and targeted therapies in oncology, advanced pancreatic cancer remains a highly morbid disease with only limited improvements in survival outcomes.^1^ According to data from the US Surveillance, Epidemiology and End Results (SEER) program, the number of incident cases of advanced pancreatic cancer was 12.5 per 100 000 per year and the number of deaths from this disease was 10.9 per 100 000 per year in 2016.^2^ Five‐year overall survival rate is consistently <10%, underscoring that advanced pancreatic cancer continues to be one of the most fatal solid tumors.^3^, ^4^ Locally advanced and metastatic pancreatic cancers are collectively referred to as *advanced pancreatic cancers* since they are managed similarly. In either clinical scenario, systemic therapy represents the main treatment modality and it is typically offered with palliative intent.

Pancreatic cancer is known for its late diagnosis, with the majority of patients presenting with unresectable disease at the time of initial diagnosis.^6^ The reason for this is likely secondary to nonspecific symptoms, such as abdominal discomfort and general malaise, which can be frequently confused with the symptoms of less serious diseases.^7^ Early detection is further complicated by the fact that pancreatic cancer often develops in elderly patients who have multiple comorbidities for which they are prescribed different medications.^8^ Therefore, it is not uncommon for symptoms of pancreatic cancer to be incorrectly attributed to these comorbidities or concomitant drugs.^9^

Following pathological diagnosis, a series of critical steps is still required to ensure optimal management, including appropriate and timely engagement of medical, radiation, surgical, and palliative oncologists. Early data suggest that some pancreatic cancer patients may not be consistently referred to specialty oncology services or managed appropriately.^10^ Possible explanations for nonreferral include patient and caregiver preferences to not pursue active anticancer therapies, poor understanding from primary care providers and other noncancer specialists about the appropriate treatment pathway, and system level barriers that prevent effective and efficient delivery of care to these patients. Further, elderly patients or those with a poor general condition may also contribute to false assumptions that oncological interventions, such as systemic therapy, are futile or may pose a high risk of toxicities without a significant degree of clinical benefit. The geographically dispersed nature of Canada and its residents can also present logistical challenges, such as a prohibitive distance between a patient's home and a tertiary cancer center where specialized pancreatic cancer care is optimally delivered.^11^

To date, the impact of referral vs nonreferral as well as the timeliness of referral, specialist consultation, and treatment initiation among advanced pancreatic cancer patients has not been characterized in detail in a population‐based setting. We hypothesized that nonreferral is prevalent and that there are inefficiencies at different time points during the management pathway. We further hypothesized that these deficiencies may result in increased use of health care services since inappropriately managed patients are more likely to experience symptoms and problems that require downstream medical attention, mainly in acute or urgent care settings.

## 2 METHODS

### 2.1 General overview of the study setting

Alberta Health Services is a provincial health authority that offers a population‐based cancer control program that is responsible for providing cancer care to approximately four million residents in the province of Alberta, Canada. At the time of this study, it was comprised of 2 tertiary, 4 regional, and 11 community cancer centers that were distributed across different catchment areas of the province to ensure equitable access to cancer care for all of its residents regardless of geography. Each tertiary and regional center offers a full range of oncological care, including ambulatory clinics, systemic therapy suites, radiation and surgical facilities, diagnostic imaging capabilities, pain and symptom management services, palliative care, and inpatient units for patients diagnosed with cancer. Radiotherapy is not delivered in the community cancer centers so patients are referred to the closest tertiary or regional center in their catchment area to receive any recommended radiation. In addition, all patients are offered opportunities to participate in oncology clinical trials. An estimated 10 000 new patients are referred to one of the cancer centers annually for management. Research ethics approval was obtained from the institutional review board prior to the conduct of this study.

### 2.2 Alberta cancer outcomes research network

The Alberta Cancer Outcomes Research Network is a provincial collaborative group of data analysts and researchers in Alberta, Canada, that conducts studies using merged data parameters extracted from the cancer registry, electronic medical records, administrative claims, and vital statistics. Data are first linked by each patient's unique lifetime identifier and then anonymized prior to analyses. The data repository contains detailed information on baseline demographics (eg, age, sex), cancer diagnosis and stage, dates of referral to oncology, clinic visits at any of the cancer centers, dates and types of therapy received for cancer, and dates of last follow‐up or death. In addition, the Charlson comorbidity index^12^ and the patterns of health care utilization can also be derived from the data using standard claims‐based algorithms that have been previously used and validated in other studies.^13^

### 2.3 Description of the study cohort and outcomes

All adult patients aged 18 years or older who were diagnosed with either stage III or IV adenocarcinoma of the pancreas from 2009 to 2016 and lived in Alberta at the time of their diagnosis were included. Because the treatment approach to pancreatic cancer differs based on histology, we excluded all patients with a diagnosis other than adenocarcinoma. The main outcomes of the study were patterns of (a) referral to cancer center (yes/no), (b) timeliness of oncology consultation (early vs late), and (c) timeliness of anticancer therapy (early vs late). Since almost all patients who were referred to a cancer center are expected to be seen by an oncologist, we opted to examine the timing of consultation (early/late) instead of the presence of a consultation (yes/no). For similar reasons, we evaluated the timing of treatment (early/late) instead. Early vs late consultations were defined as ≤14 and >14 days from the time of referral to the time of first oncology assessment, respectively, as per recent quality benchmarks outlined by several Canadian organizations.^14^ Early vs late treatments were also defined using the same 14‐day threshold as measured from the time of consultation to the time of first therapy. Sensitivity analyses were further conducted whereby we used cutoffs of 21 and 28 days. The findings were largely similar so only the main results are shown.

### 2.4 Statistical analysis

Descriptive statistics were used to summarize the baseline clinical characteristics. Groups were compared using the chi‐squared and Wilcoxon tests for categorical and continuous variables, respectively. Overall survival was analyzed with the Kaplan‐Meier method, and the log‐rank test was used to assess for significant differences among the groups. Multivariate logistic regression models were developed to determine the predictors of referral, late consultation, and late treatment, respectively, and expressed as odds ratios (ORs) and 95% confidence intervals (95% CIs). Cox regression models were subsequently constructed to generate hazard ratios (HRs) and 95% CIs for overall survival while adjusting for confounders such as age, sex, and Charlson score. All reported *P* values were two‐sided where a *P* value of <0.05 was considered statistically significant. All analyses were performed using SPSS version 20.0 (Armonk, NY, USA).

## 3 RESULTS

### 3.1 Cohort characteristics

In total, we identified 1621 eligible adult patients who were diagnosed with advanced pancreatic adenocarcinoma during the study time frame. Median age was 70 years, 50% were men, and 51% had a Charlson index of 2+. Within this cohort, only 884 (54%) patients were referred to one of the provincial cancer centers while the remaining 737 (46%) were not referred. Compared to the group that was referred, the subset that was not referred consisted of older subjects (median age 76 vs 65 years, *P* < 0.001), individuals with more comorbidities (median Charlson index 4 vs 1, *P* < 0.001), patients with prior malignancies (33% vs 24%, *P* < 0.001), and those with more advanced disease (89% vs 85%, *P* = 0.037). Additional baseline characteristics are summarized in Table 1.

**Table 1:** Characteristics of patients in the study based on referral status

Parameters	All cases (N = 1621)	Referred cases (N = 884)	Nonreferred cases (N = 737)	*P* value^a^
Age (y)				
<40	20	14 (70%)	6 (30%)	<0.0001
40‐69	759	539 (71%)	220 (29%)	
70‐80	491	250 (51%)	241 (49%)	
>80	351	81 (23%)	270 (77%)	
Sex				
Male	814	456 (52%)	428 (48%)	0.232
Female	807	358 (48%)	379 (52%)	
Charlson comorbidity score				
0	576	352 (62%)	224 (38%)	<0.0001
1	157	98 (63%)	59 (37%)	
2	53	26 (49%)	27 (51%)	
2+	835	408 (49%)	427 (51%)	
Prior malignancy				
No	1173	673 (57%)	500 (43%)	<0.0001
Yes	448	211 (47%)	237 (53%)	
AJCC stage				
III	210	129 (61%)	81 (39%)	0.037
IV	1411	755 (54%)	656 (46%)	
Location				
Head	649	364 (56%)	285 (44%)	0.372
Body	261	138 (53%)	123 (47%)	
Tail	306	155 (51%)	151 (49%)	
Overlapping	246	143 (58%)	103 (42%)	
Unspecified	159	84 (53%)	75 (47%)	
Causes of death				
Pancreatic cancer	1431	763 (53%)	668 (47%)	<0.0001
Other cancer	9	4 (44%)	5 (56%)	
Noncancer	97	68 (70%)	29 (30%)	
Alive	84	49 (58%)	35 (42%)	

AJCC, American Joint Committee on Cancer; % = row percentages.

a Chi‐square test was used.

### 3.2 Patterns of referral, consultation, and treatment

Among patients who were referred to a cancer center and subsequently proceeded to an assessment by an oncologist (N = 816), the median time from referral to consultation was 16 (IQR 8‐27) days. Based on our a priori decision to use the 14‐day benchmark as the cutoff between early and late, 55% of patients were considered to have undergone a *late* evaluation by a cancer specialist. Only 410 (50%) of those assessed by an oncologist were given anticancer therapy. In this treated group, the median time from consultation to initiation of therapy was 17 (IQR 8‐36) days. Treatment included mainly of systemic therapy (95%) followed by palliative radiotherapy (9%) and palliative surgical intervention (2%). Some patients received more than one treatment modality over the course of their advanced disease. No differences in baseline characteristics were observed when comparing patients with early vs late consultation times. Comparing early vs late treatment times, patients with more advanced disease were more likely than those with less advanced disease to start therapy early (90% vs 79%, *P* = 0.003). Otherwise, there were no differences between groups.

In multivariate logistic regression models (Table 2), specific baseline clinical factors were associated with referral patterns to a cancer center. For example, younger age (OR of referral, 3.76, 95% CI: 2.74‐5.15, for ages 70‐80 years vs >80 years) and lower comorbidity burden (OR of referral, 1.80, 95% CI: 1.41‐2.31, for Charlson 0 vs >2) predicted for referral whereas older age and higher comorbidity levels predicted for nonreferral. In addition, pancreatic head tumors when compared to other tumor locations were associated with longer intervals from referral to oncology consultation (OR of late consultation, 0.53, 95% CI: 0.34‐0.83, for pancreatic body vs head). Moreover, specific factors were also associated with prolonged consultation to treatment, including lower comorbidity scores (OR of late treatment, 2.00, 95% CI: 1.32‐3.02, for Charlson score 0 vs score >2) and earlier disease stage (OR of late treatment, 0.49, 95% CI: 0.26‐0.96 for stage IV vs stage III). Notably, the year of diagnosis also impacted referral to consultation times whereby patients diagnosed in more recent years were less likely to wait beyond 14 days to see an oncologist (OR of late consultation, 0.84, 95% CI: 0.77‐0.91).

**Table 2:** Multivariate models of factors associated with referral, late consultation, and late treatment

Parameters	Referral		Late consultation		Late treatment	
	OR (95% CI)	*P* value	OR (95% CI)	*P* value	OR (95% CI)	*P* value
Age (y)						
>80	Reference		Reference		Reference	
<40	13.57(4.69‐39.26)	<0.0001	1.80 (0.49‐6.57)	0.375	1.57 (0.26‐9.56)	0.624
40‐69	9.22 (6.74‐12.63)	<0.0001	1.60 (0.91‐2.82)	0.101	2.91 (0.86‐9.91)	0.087
70‐80	3.76 (2.74‐5.15)	<0.0001	1.43 (0.79‐2.59)	0.234	2.18 (0.61‐7.83)	0.232
Sex						
Male	Reference		Reference		Reference	
Female	0.98 (0.80‐1.20)	0.846	1.19 (0.88‐1.61)	0.263	1.21 (0.78‐1.87)	0.395
Charlson comorbidity score						
2+	Reference		Reference		Reference	
0	1.80 (1.41‐2.31)	<0.0001	1.26 (0.90‐1.77)	0.178	2.28 (1.42‐3.66)	0.001
1	1.94 (1.31‐2.86)	0.001	1.11 (0.67‐1.84)	0.677	2.72 (1.25‐5.90)	0.011
2	1.75 (0.93‐3.29)	0.082	0.77 (0.31‐1.92)	0.579	2.16 (0.54‐8.55)	0.274
Prior malignancy						
No						
Yes	1.14 (0.86‐1.49)	0.368	1.44 (0.95‐2.17)	0.087	1.62 (0.88‐2.99)	0.124
AJCC stage						
III	Reference		Reference		Reference	
IV	0.78 (0.56‐1.09)	0.141	0.91 (0.59‐1.39)	0.653	0.49(0.26‐0.96)	0.036
Location						
Head	Reference		Reference		Reference	
Body	0.82 (0.60‐1.13)	0.228	0.53 (0.34‐0.83)	0.005	1.04 (0.53‐2.03)	0.919
Tail	0.71 (0.52‐0.97)	0.030	0.83 (0.54‐1.28)	0.398	0.65 (0.35‐1.21)	0.176
Year of diagnosis (continuous variable)	1.05 (0.98‐1.11)	0.107	0.84 (0.77‐0.91)	<0.0001	0.89 (0.79‐1.01)	0.056

### 3.3 Survival and health services outcomes

The median overall survival was 4.7 and 1.0 months between referred and nonreferred groups, 4.6 and 5.6 months between early and late consultation groups, and 6.4 and 8.9 months between early and delayed treatment groups (all *P* < 0.05). Pancreatic cancer was the cause of death in the majority of cases (88%).

A multivariable Cox proportional hazard model was conducted for predictors of overall survival among patients who were treated (Table 3). The following factors were associated with longer overall survival: younger age (HR for death, 0.51, 95% CI: 0.30‐0.86 for ages 70‐80 years vs >80 years), earlier disease stage (HR for death, 0.60, 95% CI: 0.47‐0.79 for stage III vs stage IV), and fewer comorbidities (HR for death, 0.70, 95% CI: 0.57‐0.86 for Charlson 0 vs >2). Of note, neither referral to consultation time nor consultation to treatment interval correlated with overall survival. Finally, we compared acute health care services use, such as the frequency of emergency room visits and hospital admissions per survival time, as a proxy measure of symptom crisis requiring acute medical attention secondary to the underlying pancreatic cancer. Compared to referred patients, those who were not referred to the cancer center experienced more overall acute care use. For example, nonreferred patients had 13.4 hospitalization days compared to 5.6 hospitalization days for referred patients (Table 4).

**Table 3:** Multivariate model of factors associated with overall survival in treated patients

Parameters	HR (95% CI)	*P* value
Age (y)		
>80	Reference	
<40	0.27(0.11‐0.64)	0.004
40‐69	0.38 (0.22‐0.66)	0.001
70‐80	0.43 (0.24‐0.77)	0.005
Sex		
Female	Reference	
Male	1.09 (0.89‐1.35)	0.398
Charlson comorbidity score		
2+	Reference	
0	0.77 (0.61‐0.97)	0.026
1	0.61 (0.42‐0.88)	0.007
2	0.36 (0.17‐0.74)	0.006
AJCC stage		
IV	Reference	
III	0.75 (0.65‐0.87)	<0.0001
Location		
Head	Reference	
Body	1.26 (0.92‐1.73)	0.145
Tail	1.30 (0.95‐1.77)	0.093
Referral to consultation time		
≤14 d (early)	Reference	
>14 d (late)	0.81 (0.66‐1.02)	0.065
Consultation to treatment time		
≤14 d (early)	Reference	
>14 d (late)	0.86 (0.69‐1.17)	0.184

**Table 4:** Comparison of health services use^a^ between referred and nonreferred patients

Type of health services	Referred patients	Nonreferred patients	*P*‐value^b^
	Mean (SD) encounters	Mean (SD) encounters	
Duration of all hospital admissions (d)	5.56 (6.48)	13.41 (33.80)	<0.0001
Number of primary care visits	7.69 (7.35)	13.80 (10.35)	<0.0001
Number of general surgeon visits	8.03 (28.60)	11.63 (35.76)	0.024
Number of gastroenterologist visits	4.25 (12.69)	11.90 (38.19)	<0.0001
Number of emergency visits	3.80 (7.32)	3.50 (10.73)	0.507

a Adjusted by dividing encounters by years of survival.

b Independent *T* test.

## 4 DISCUSSION

The current study evaluates patterns of referral, consultation, and treatment of patients with advanced pancreatic cancer in a population‐based setting. Overall, it demonstrated that close to half of patients were not referred to a cancer center for consultation, which was especially evident among older patients as well as those with multiple comorbidities. Referred patients who were diagnosed in more recent years were less likely to experience late consultation even though intervals from referral to consultation and from consultation to treatment did not appear to impact overall survival. Importantly, nonreferred patients were more likely to experience specific acute care encounters, particularly longer total duration of hospitalizations and more frequent visits to specific physician specialists.

One of the noteworthy features of this study is our ability to examine referral patterns. Most prior institutional series were unable to evaluate this because it is often difficult to reliably ascertain the referral base (eg, denominator).^15^ The population‐based setup of the cancer care delivery system in Alberta where all malignant diagnoses are recorded and where referrals to any cancer center in the province are captured enables us to study referral patterns. While it is not surprising that older and frailer patients were less likely to be referred to see an oncologist, the large proportion of nonreferred patients (46%) is unexpected. Gemcitabine monotherapy was the standard of care for over 15 years until as recently as 2012 when newer regimens were introduced.^4^, ^5^ Gemcitabine was not associated with a significant survival advantage albeit it may offer some clinical benefits (eg, lower pain, better appetite, and less weight loss).^16^ Thus, one possible reason for the nonreferral could be the lack of awareness among primary care providers of more effective treatment options that recently became available. Their assumptions that only gemcitabine was available and that treatments would be unlikely to alter the overall trajectory of the disease may have resulted in nonreferral to oncologists in favor of early engagement of community palliative care or hospice care as the more appropriate action.

Of note, neither long referral to consultation times nor long consultation to treatment times negatively impacted prognosis. However, this should be interpreted cautiously since timing of therapy has been shown to be clinically relevant in other malignant settings (such as breast and colorectal cancers) where delays beyond a certain threshold seem to pose a detrimental effect on survival.^17^, ^18^ The reason that a similar relationship was not observed in advanced pancreatic cancer is unclear, but it is possible that timing of therapy represents a more significant modifier of outcomes when the biology and natural history of the disease is more indolent and chronic as is the case for many breast and colorectal cancer where patients can live for many years. In our study cohort, more than half of patients experienced a relatively short interval between referral date and oncology assessment as well as a brief interval between oncology consultation and treatment initiation (16 and 17 days, respectively). This may have further weakened any associations that would have been observed if more patients were to have experienced longer delays.

Moreover, we examined downstream effects to the healthcare system by comparing acute care encounters between referred and nonreferred patients with advanced pancreatic cancer. In addition to delivering active anticancer therapies to patients, oncologists also play a significant role in a patient's overall well‐being by managing their symptoms, providing emotional and psychological support, and referring them to palliative care when appropriate. Because many of these important aspects of care are difficult to measure directly, we used encounters with acute care services as a proxy. This is a reasonable surrogate marker since patients typically present urgently to medical attention when symptoms are poorly managed, when supports at home are inadequate, or when end‐of‐life care needs are not sufficiently addressed.^19^ In our analysis, we found that nonreferred patients are more likely to use specific acute care services, such as hospitalizations, when compared to referred patients. Despite the potential for confounding by indication, this finding can also be interpreted as suggesting that all patients may benefit from a referral to a cancer center regardless of whether or not they ultimately receive active anticancer therapy.

There are several limitations to this study. First, this was a retrospective analysis of mainly registry and administrative data. Therefore, selection bias is inherent to the study design. However, our conclusions are largely based on multivariate models that adjusted for as many measured confounders as possible in order to minimize the effects of confounding. Nevertheless, the risk of residual confounding from unmeasured factor remains concerning. Second, we observed that advanced age and frailty from comorbidities were predictors of nonreferral. However, the decision to forgo referral is likely significantly more complex and can be driven by patient choice, physician discretion, and other situational factors. Understanding these aspects in detail would require a qualitative study that is beyond the scope of the current work. Third, the data did not capture patients with a new diagnosis of pancreatic cancer in whom an inpatient consultation was conducted, but we expect the number of affected patients to be low. Fourth, several factors that may influence the likelihood of consultation and treatment were not available in the study dataset, such as performance status and socioeconomic status. The limited sample size may have further restricted the representativeness of our conclusions. These limitations, however, should be considered in the context of the study's many strengths, which include its relatively large cohort size, population‐based design, and longitudinal time frame of 8 years. It also represents one of the few studies that can accurately investigate referral patterns because the Alberta cancer care system captures all malignant diagnoses and oncology clinic visits that occur in the province.

In summary, a significant proportion of patients with advanced pancreatic cancer in this population‐based cohort were never referred to a cancer center. Once they were referred, however, the majority of patients were quickly assessed by an oncologist and received treatment in approximately 2 weeks, potentially explaining why the time intervals from referral to consultation and from consultation to treatment were not correlated with outcomes. Nonreferred patients experienced increased health services encounters, suggesting that oncologists may contribute to the management of symptoms that reduce the need for acute care. Efforts to understand some of the drivers of nonreferral may help to improve the overall quality of care that is being provided to advanced pancreatic cancer patients.

## ETHICS APPROVAL

As this was a secondary analysis of administrative data with no direct contact with patients, it was considered minimal risk and informed consent was waived.

## CONFLICT OF INTEREST

None declared.
